# Heterotopic Cutaneous Adnexa Arising from the Ascending Colon Presenting as a Chronic Abdominal Wall Sinus: A Rare Congenital Anomaly and Diagnostic Challenge

**DOI:** 10.3390/diagnostics16193251

**Published:** 2026-10-08

**Authors:** Gang Xiao, Dan-Dan Li, Qiu-Shi Huang, Shan He, Yuan Liang, Jian Shen

**Affiliations:** 1 Department of General Surgery, Sichuan University Affiliated Chengdu Second People’s Hospital, Chengdu Second People’s Hospital, West China School of Medicine, Sichuan University, Chengdu 610017, China; xiaogang@sc-renji.cn (G.X.); huangqs0419@163.com (Q.-S.H.); hes2026@163.com (S.H.); 2 Department of Orthopedics, Sichuan University Affiliated Chengdu Second People’s Hospital, Chengdu Second People’s Hospital, West China School of Medicine, Sichuan University, Chengdu 610017, China; lidandan20221110@hotmail.com; 3 Department of Pathology, Sichuan University Affiliated Chengdu Second People’s Hospital, Chengdu Second People’s Hospital, West China School of Medicine, Sichuan University, Chengdu 610017, China; liangy200419@163.com

**Keywords:** heterotopic cutaneous adnexa, ascending colon, chronic abdominal wall sinus, congenital developmental anomaly, ectodermal remnant, laparoscopic surgery

## Abstract

A chronic abdominal wall sinus tract caused by heterotopic cutaneous adnexa arising from the colonic serosa is extremely rare and readily misdiagnosed. We report a 39-year-old woman with congenital scoliosis and a 30-year history of intermittent, odorless, non-feculent discharge from a cutaneous opening in the right lower abdominal wall; she had no prior abdominal surgery. Contrast-enhanced computed tomography (CT) revealed a non-enhancing sinus tract extending from the ascending colon toward the abdominal wall, whereas colonoscopy showed normal colonic mucosa with no fistulous communication. Laparoscopic exploration revealed a tubular structure arising from the serosal surface of the middle ascending colon that bifurcated into an abdominal wall branch and a pelvic branch, the latter not identified on preoperative imaging. Because the lesion was considered benign but preoperative imaging could not determine the depth of involvement, and an unexpected pelvic branch was found intraoperatively, a laparoscopic limited right hemicolectomy was performed with en bloc excision of both branches. Histopathological examination confirmed the absence of intestinal epithelial components, and immunohistochemistry was positive for cytokeratin 7 (CK7), GATA-binding protein 3 (GATA-3), and p63, confirming a cutaneous adnexal origin. The patient recovered uneventfully, with no recurrence at the 6-month follow-up. This case may represent a rare congenital developmental anomaly related to abnormal persistence of ectodermal components during embryologic development; however, definitive embryologic proof is lacking. It emphasizes the importance of a detailed history, the inclusion of congenital heterotopic cutaneous adnexal lesions in the differential diagnosis of refractory abdominal wall sinus tracts, and complete excision of all sinus tract branches to prevent recurrence and repeated ineffective interventions.

**Figure 1:**
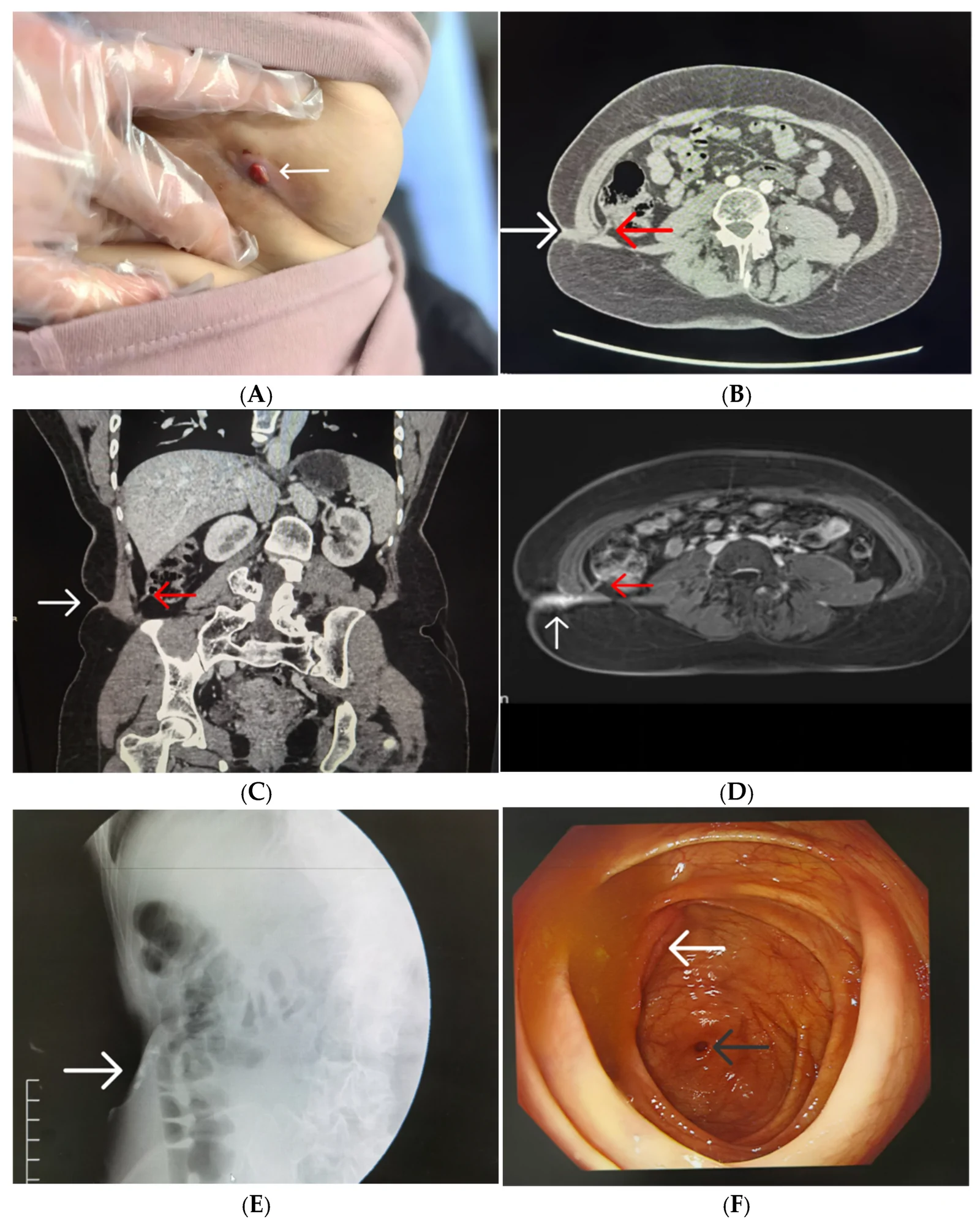
Clinical presentation and imaging findings of a chronic abdominal wall sinus tract. A 39-year-old woman with congenital scoliosis was admitted on 24 February 2026 with a 30-year history of intermittent, odorless, non-feculent discharge from a cutaneous opening in the right lower abdominal wall. Physical examination revealed fresh granulation tissue and pigmentation around the opening, without redness, swelling, or tenderness (**A**, white arrow), and she had no history of abdominal surgery. Contrast-enhanced CT revealed a non-enhancing subcutaneous tract extending from the abdominal wall into the abdominal cavity and closely adjacent to the ascending colon. Axial contrast-enhanced CT (**B**) showed this tract (white arrow) extending from the abdominal wall into the abdominal cavity, closely adjacent to the ascending colon (red arrow); coronal reconstructed contrast-enhanced CT (**C**) showed the same tract (white arrow) and the ascending colon (red arrow). Contrast-enhanced magnetic resonance imaging (MRI) (**D**) demonstrated a small patchy lesion with prolonged T1 and T2 signal intensity in the right abdominal wall (white arrow), with an enhancing cord-like structure extending into the abdominal cavity (red arrow). Sinography showed that the contrast agent remained within the subcutaneous tissue (white arrow, **E**) and did not enter the ascending colon. Colonoscopy demonstrated a normal appendiceal orifice (black arrow, **F**) and ileocecal valve opening (white arrow, **F**), without colonic inflammation, ulceration, or fistulous communication. Retrospective review of the preoperative CT and MRI did not reveal a definite pelvic branch, which was likely collapsed and non-enhanced, and was therefore not identified before surgery.

**Figure 2:**
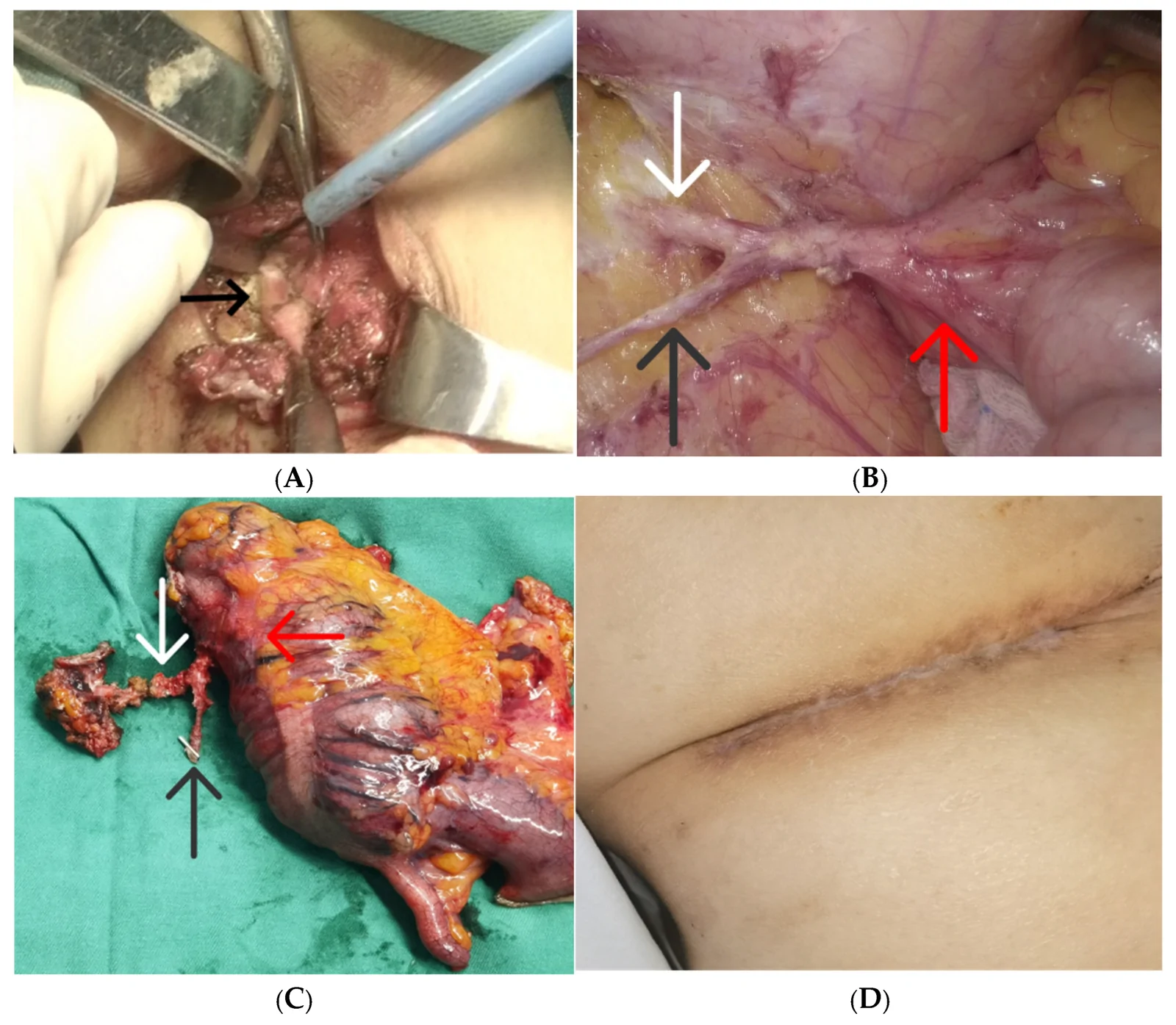
Intraoperative findings and postoperative outcome. On 28 February 2026, the patient underwent surgery. Through a fusiform incision (**A**), the tubular structure located between the skin, the abdominal wall, and the surrounding tissues was dissected and removed en bloc (black arrow). Laparoscopic exploration (**B**) revealed that the structure originated from the serosal surface of the middle segment of the ascending colon (red arrow); one branch extended toward the abdominal wall (white arrow), whereas an unexpected second branch extended into the pelvic cavity (black arrow) and had not been identified on preoperative imaging. Although preoperative imaging suggested a benign lesion, it could not determine whether the lesion was confined to the serosal surface or involved deeper layers. Because the colonic attachment was broad-based and intimately associated with the ascending colon, and because complete excision of all branches was considered essential to prevent recurrence, the patient underwent laparoscopic limited right hemicolectomy with en bloc removal of the entire sinus tract system. A simple wedge resection was considered but was judged to carry a higher risk of incomplete resection and recurrence, as it might not ensure complete removal of all branches or adequate margins. The patient was fully informed and consented to this approach. The resected specimen (**C**) showed the tubular structure connecting the colonic serosa (red arrow) with the abdominal wall muscle and skin (white arrow), with a branching configuration (black arrow). At the 6-month follow-up, the original sinus tract wound had completely healed (**D**).

**Figure 3:**
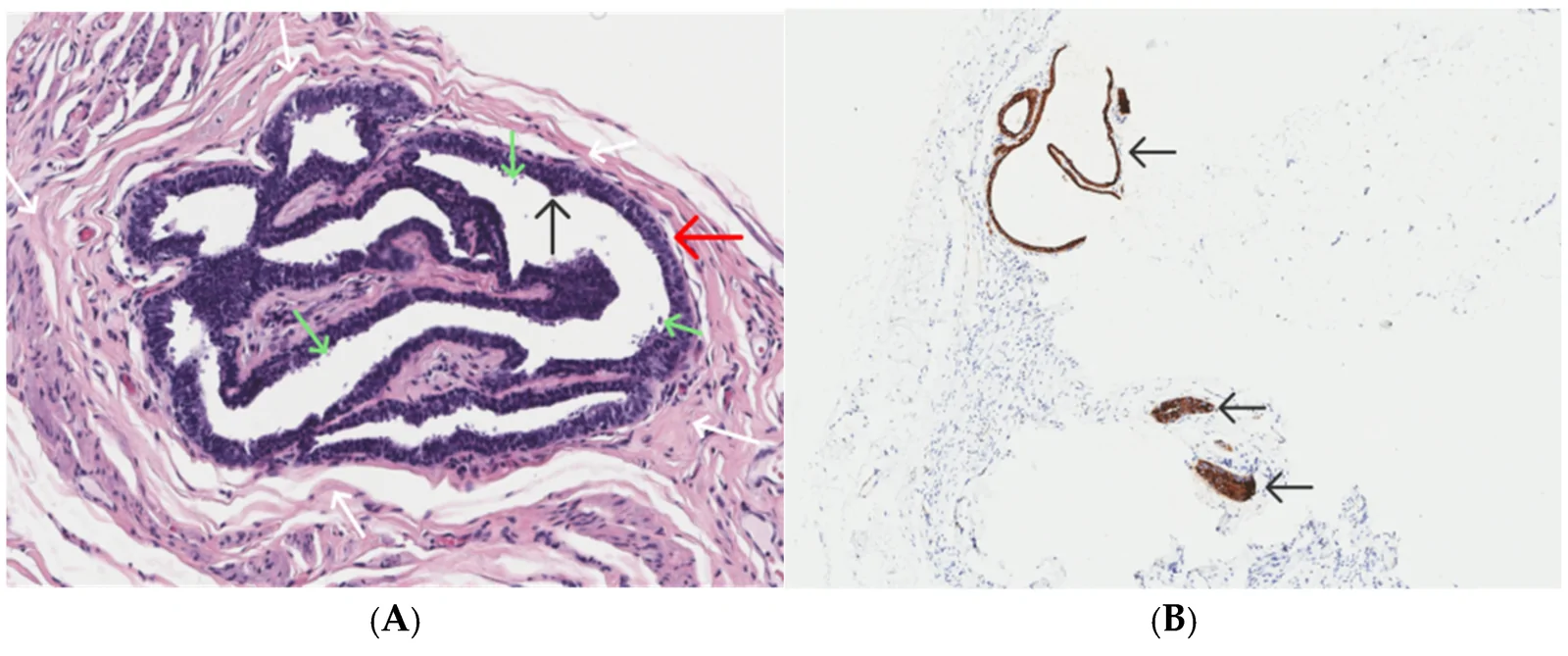

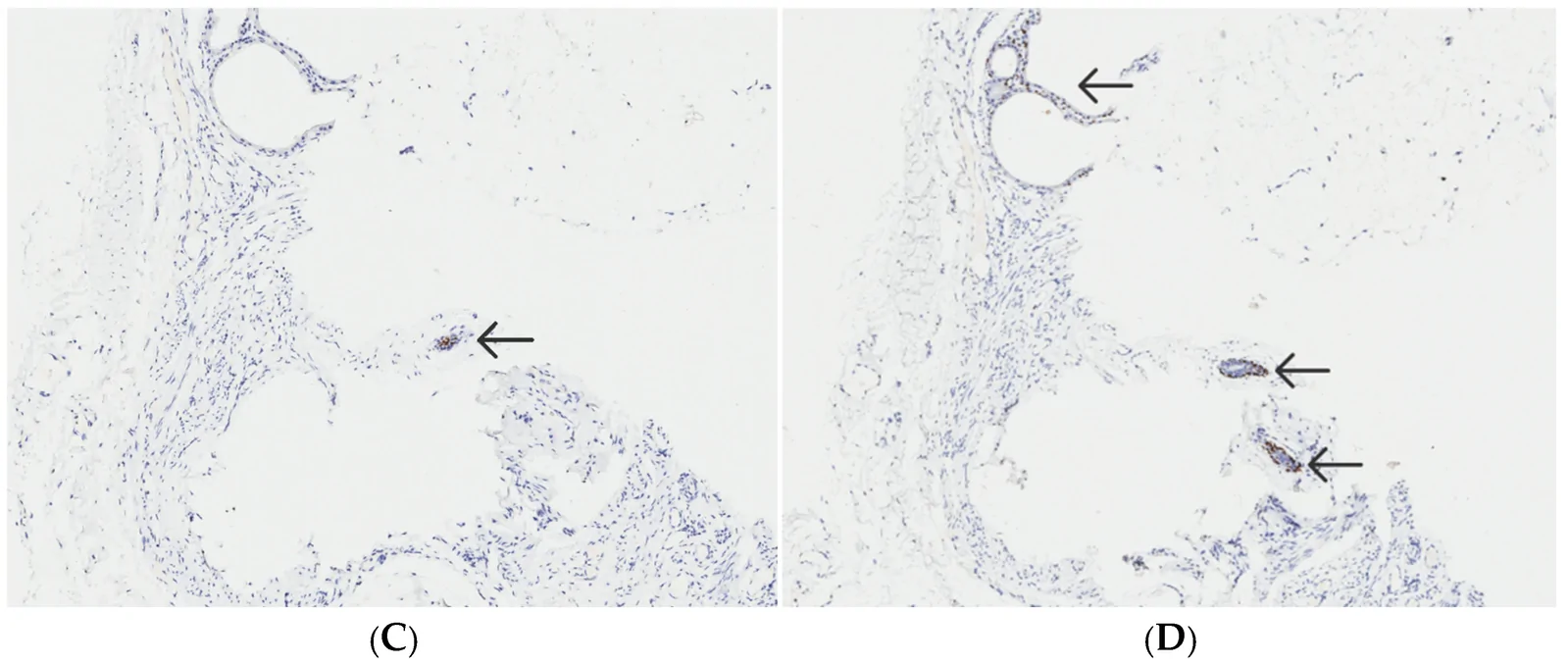
Histopathological and immunohistochemical findings. Histopathological examination (**A**) with hematoxylin and eosin staining (×200) showed irregular tubular structures (white arrows) within nodularly hyperplastic smooth muscle tissue; the inner layer consisted of cuboidal/columnar epithelium (black arrows) with focal secretory activity (green arrows), and the outer layer was composed of squamous epithelium (red arrows). No intestinal epithelial components were identified. Immunohistochemical analysis (×400) showed that the inner epithelial cells were positive for cytokeratin 7 (CK7) (**B**, arrows) and GATA-binding protein 3 (GATA-3) (**C**, arrows), whereas the outer epithelial component expressed p63 (**D**, arrows), confirming a cutaneous adnexal origin (**B**–**D**) (400×). Chronic abdominal wall sinus tracts typically present as persistent, recurrent, or non-healing cutaneous openings. Their formation is a complex process involving infection, foreign-body reactions, and chronic inflammation, and they most commonly follow postoperative infection or retained foreign material [1,2]. Persistent discharge may be purulent or serous; an odorless discharge usually suggests chronic inflammation or a foreign-body reaction, whereas malodor may indicate a specific infection or enteric communication. Long-term irritation may cause eczematous skin changes, pigmentation, or scar hyperplasia around the opening. Importantly, imaging plays a central role in the evaluation of abdominal wall sinus tracts. CT effectively delineates the extent of the sinus tract and identifies potential fistulous communication, and it offers advantages over plain radiography in defining tract extent and associated fistula formation [3,4]. However, as illustrated by our case, small or collapsed branches may remain undetected on preoperative imaging; careful intraoperative exploration and complete excision of all branches are therefore essential. Before attributing such a tract to a congenital cause, acquired causes should be carefully excluded. Enterocutaneous fistulas communicate with the bowel lumen, often produce feculent or enteric discharge, and are associated with Crohn’s disease [5], tuberculosis, malignancy, or prior surgery [1,2]; in our patient, colonoscopy and sinography showed no fistulous communication. Dermoid/epidermoid cysts and pilonidal sinuses [6,7] are lined by squamous epithelium and generally lack a connection to the colonic serosa; dermoid cysts may contain adnexal structures but are typically cystic rather than tubular branching tracts. Postoperative sinus tracts follow prior surgery, retained foreign bodies, or spilled gallstones, none of which applied to our patient. Together, these features made a congenital heterotopic cutaneous adnexal remnant the most plausible diagnosis. The histopathological features observed here were distinctly different from those of dermoid cysts and pilonidal sinuses, which typically lack organized cutaneous adnexal structures [4,8]. The positive immunohistochemical expression of CK7, GATA-3, and p63 strongly supported a cutaneous adnexal origin. Together with the intraoperative finding of a tract connecting the abdominal wall to the colon and the coexistence of congenital scoliosis, these findings favor a congenital developmental origin. However, because direct embryologic evidence is unavailable, this mechanism remains a hypothesis. From an embryologic perspective, the skin and its appendages derive from the ectoderm [9], whereas the colonic serosa derives from the mesoderm, and a direct developmental connection between these tissues has not been established. During early gastrulation, however, the ectoderm and mesoderm are closely apposed and interact through reciprocal signaling; abnormal separation, adhesion, or migration of ectodermal cells into the midgut mesenchyme could theoretically give rise to heterotopic cutaneous adnexa on the colonic serosa. The coexistence of congenital scoliosis, itself a developmental anomaly, may suggest a broader disturbance of early embryonic development, although this remains speculative. Alternatively, the lesion may represent heterotopic differentiation of multipotent mesenchymal cells, but the immunohistochemical profile supports a cutaneous adnexal origin [10]. We therefore present the embryologic mechanism as a hypothesis rather than a definitive conclusion. Complete surgical excision remains the mainstay of treatment and is essential to prevent recurrence [8]. In our patient, complete excision of all sinus tract branches required laparoscopic limited right hemicolectomy because of the broad-based colonic attachment and the unexpected pelvic branch; moreover, preoperative imaging suggested a benign lesion but could not determine whether it was confined to the serosal surface or involved deeper layers. This approach achieved en bloc removal with adequate margins and ensured complete resection. In summary, this case represents a rare colonic–cutaneous sinus tract with distinctive clinical and pathological characteristics, providing valuable insight into the diagnosis and management of similar challenging cases. When evaluating patients with chronic, sterile, non-enteric abdominal wall sinus tracts, particularly those with features suggestive of a developmental anomaly such as congenital scoliosis, clinicians should consider the possibility of congenital ectodermal remnants. This consideration may prevent prolonged misdiagnosis and facilitate timely definitive resection. Nevertheless, acquired causes, including enterocutaneous fistulas associated with Crohn’s disease [3], tuberculous sinus tracts, and postoperative foreign-body granulomas [1,2], should be carefully excluded. Complete surgical excision remains the mainstay of treatment and is essential to prevent recurrence [8].

## Data Availability

The raw data supporting the conclusions of this article will be made available by the authors on request.

## Abbreviations

The following abbreviations are used in this manuscript:

CT	Computed tomography
MRI	Magnetic resonance imaging
CK7	Cytokeratin 7
GATA-3	GATA-binding protein 3
